# What is the total impact of an obstetric anal sphincter injury? An Australian retrospective study

**DOI:** 10.1007/s00192-019-04108-3

**Published:** 2019-09-16

**Authors:** Elizabeth Evans, Clorinda Falivene, Kathy Briffa, Judith Thompson, Amanda Henry

**Affiliations:** 1 School of Physiotherapy and Exercise Science, Curtin University, Perth, Western Australia Australia; 2 Physiotherapy Department, Royal Hospital for Women, Barker Street, Sydney, New South Wales 2031 Australia; 3 School of Women’s and Children’s Health, UNSW Medicine, Sydney, New South Wales Australia; 4 Women’s and Children’s Health, St George Hospital, Sydney, New South Wales Australia

**Keywords:** OASI, Perineal tear, Birth injury, Third degree tear, Anal incontinence, Dyspareunia

## Abstract

**Introduction:**

Most data on obstetric anal sphincter injury (OASI) reflect short-term (< 12 months) or much longer term (> 10 years) impact. This study aimed to collate the extent of medium-term symptomology (1–6 years) and observe the effect on future birth choices to evaluate the cumulative impact of OASI in affected women.

**Methods:**

A retrospective cohort of women affected by OASI completed a questionnaire covering bowel symptomology, sexual function, life impact and future birth choices. A custom-created adverse composite outcome for OASI incorporating effects on daily life, flatal/fecal incontinence and sexual function (OASIACO) was used as a threshold score to identify women with high levels of symptoms.

**Results:**

Of 265 eligible and contactable women, 210 questionnaires were received (response rate 79%) at a mean of 4 years post-OASI. More than half (54%) experienced an OASIACO. A forceps birth (*p* = 0.03) or more severe grade of tear (*p* = 0.03) was predictive of OASIACO. One hundred one women had further children, with 48% reporting their delivery choices were impacted, 32% electing a cesarean delivery and 26% shifting to private care. Eighty women (40%) had not given birth again, and 29 (36%) of these indicated their OASI influenced this decision.

**Conclusions:**

The total impact of an OASI on women affected is substantial. More than half experience ongoing symptoms and close to half report an impact on their future birth choices. It follows there would be a consequential load on the healthcare sector, and improved management and prevention programs should be implemented.

**Electronic supplementary material:**

The online version of this article (10.1007/s00192-019-04108-3) contains supplementary material, which is available to authorized users.

## Introduction

An obstetric anal sphincter injury (OASI) is defined as a partial or complete disruption of the anal sphincter muscles, sustained during childbirth, which includes either or both the internal and external anal sphincter. Also referred to as third and fourth degree tears, they may be classified according to their severity (Appendix A1).

It is well documented that an OASI is a major factor in the development of anal incontinence, a distressing symptom that can be devastating to the woman affected [1, 2]. Anal incontinence refers to the involuntary loss of solid or liquid stool or of gas. The prevalence of reported anal incontinence in women after an OASI varies between study populations, ranging from 15 to 61% [3, 4]. Poor quality of life and high morbidity associated with OASI are commonly reported by women, with dyspareunia, perineal pain and urinary incontinence the most common [5, 6]. At the time of this research there were very limited medium to longer term studies reporting the persistence of symptoms, few that took into account the spectrum of symptomology experienced by the whole woman and none within the Australian population [3–7].

The unexpected morbidity experienced by some women following OASI significantly impacts the many challenges women face in the transition to motherhood, making the postnatal period increasingly stressful and complex [8]. To optimize OASI management pathways, greater insight into the true extent of symptoms, and their time course, is required. It is hoped that with respectful health support of women with OASI, the ongoing symptomology, postnatal distress and medicolegal claims, driven by women feeling distraught or neglected, may be minimized [9]. The NHS Litigation Authority 10-year report on maternity claims identified perineal trauma as the fourth highest indication for claims [10].

The primary aims of this study were: (1) to determine the women’s reported symptomology and daily life impact in the medium term after an OASI (from 16 months to 6.5 years) and (2) to document the impact of OASI on women’s future birth choices.

## Materials and methods

This was a retrospective cohort of women identified as having been diagnosed with an OASI at the Royal Hospital for Women (RHW), Sydney, Australia. RHW is a tertiary maternity hospital managing approximately 4000 births per year with an OASI clinic in operation.

Potential participants were women, giving birth as a ‘public patient’ (not under the direct care of a private obstetrician: public care being the predominant model of maternity care in Australia generally and the study hospital), identified on 21 April 2015 from the hospital database (Obstetrix Consortium, NSW Health) as being diagnosed with an OASI in the recruitment time period from October 2009 to May 2014.

Inclusion criteria for this study were:

1. Women giving birth as a public patient during the study time frame;
2. An OASI identified at the time of this birth and recorded on the hospital’s database;
3. Women at least 13 months post-injury when completing the questionnaire;
4. Contact details available in the woman’s medical record.

Women were excluded if they were unable to read, write or understand basic English or if their birth involved neonatal death or intrauterine fetal death (stillbirth).

Eligible women were sent an invitation letter via post with a hard copy of the study questionnaire, a consent form and a stamped return envelope for the completed questionnaire. An online version of the questionnaire was also created using a secure server as an alternate option if preferred. Online completion of the questionnaire was taken as consent to participate.

The questionnaire (Appendix A2) consisted of three main sections: the previously validated Manchester Health Questionnaire [11], the sexual function section of the validated Australian Pelvic Floor Questionnaire [12] and a number of custom-designed questions seeking to illuminate the woman’s health care experience and impact on subsequent birth choices as well as an open comment qualitative section.

Our primary outcome measure was a custom-created ‘adverse composite outcome’ (OASIACO), a threshold score to encapsulate unacceptable levels of ongoing symptomatology as a result of an OASI. These categories were decided upon by the authors based on discussions with women, health workers and review of the literature for symptomology that the average person would deem unacceptable to live with.

OASI questionnaire respondents were considered to have an OASIACO if any of the following criteria were met:

1. The OASI had a reported impact on daily life in at least three out of six specified areas: physical impact, emotional impact, sexual impact, ability to return to work, ability to exercise or ability to do normal activities;
2. Reported symptoms of flatal incontinence or fecal urgency “sometimes” or more often, or symptoms of fecal incontinence (of loose or solid stool) occurring “occasionally” or more often (rated on a five-point Likert scale);
3. Achieving a score > 5.04/21 (raw score) indicating a high level of sexual dysfunction on the Australian Pelvic Floor Questionnaire section [12].

The OASIACO was further analyzed to recognize the dimension of severity of symptoms: identifying the proportion of women reporting one, two and three components of the OASIACO (OASISACO1, OASIACO2, OASIACO3), respectively. Secondary outcomes included presence of individual components of the OASIACO and stated effect of the OASI on women’s future birth choices.

### Statistical analyses

Data were collated and then analyzed using the IBM SPSS Statistics Package 24 (SPSS Statistics for Windows, IBM, Armonk, NY). Demographic variables, obstetric outcomes (including degree of OASI) and rates of OASIACO and its subcomponents were descriptively analyzed and presented as mean ± standard deviation for continuous data (or median/interquartile range if non-normally distributed) and as number (percentage) for categorical data. The demographic, obstetric and level of support variables for women with OASIACO (the primary outcome) were compared with those without OASIACO, using the t-test for normally distributed continuous data, Mann-Whitney U testing for non-normally distributed continuous data and chi-squared or Fisher’s exact testing as appropriate for categorical data. The demographic and obstetric characteristics of participating women were also compared with those of non-participating women to assess to what degree survey data were likely to have been affected by respondent bias. A *p* value < 0.05 was considered statistically significant; biological plausibility was considered to ensure findings were not by chance.

### Ethical approval

The study was granted ethical approval by the South-Eastern Sydney Local Health District Human Research Ethics Committee (14/165) and Curtin University, Perth (HR49/2015). Original approval was granted 22 September 2014 and amendment approval on 23 April 2015.

## Results

**Fig. 1:**
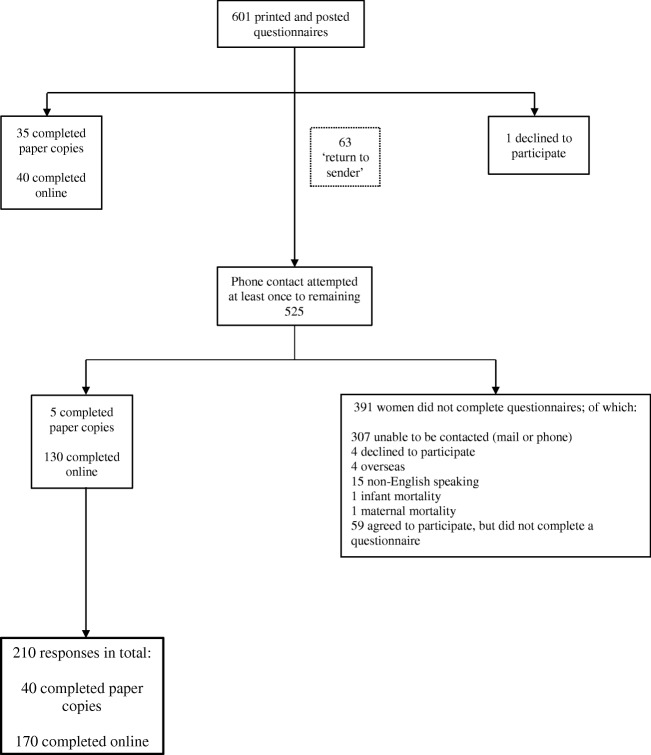
Flowchart of participant recruitment and study population

Six hundred one women were identified (affected by 604 OASI incidents) during the study period, giving an overall OASI rate of 4.6% across this time period. A flow diagram of participant recruitment is outlined in Fig. 1. The response rate when considering the number of respondents as a proportion of the total number of women affected by an OASI in the specified time period was 210/601 (35%). However, when considering the number of women meeting the eligibility criteria and with whom direct contact and invitation to participate were able to be made (265 women), the response rate was 210/265 (79%). Of the 210 respondents, 200 completed all sections of the questionnaire to allow presence or absence of the OASIACO to be calculated. Women responding to the questionnaire were, on average, 4 years since OASI birth, range 16–79 months. The obstetric characteristics of the 210 respondents did not differ considerably from those of the women who did not complete the questionnaire; however, non-respondents were slightly younger at the time of the affected birth and more likely to be overseas-born (Table 1).

**Table 1:** Characteristics and birth data of OASI respondents versus non-respondents and overall hospital maternity population during the study period. Bold *P* values indicate statistical significance *P*<0.05

		Respondents *n* = 210	Non-responders *n* = 393	*P* value respondents versus non-respondents	Hospital average 2009–2014^1^ 24,576	*P* value total population versus OASI respondents
Mean age at time of birth (years)		31.6	30.5	**0.003**	32.4	**0.003**
Gestation at birth (weeks)		40.0	40.0	0.82	38.8	**< 0.001**
Parity^2^	0 1 2+	81% 17% 2%	83% 15% 2%	0.49 0.44 1.0	55% 31% 14%	**< 0.001** **< 0.001** **< 0.001**
Australian-born		40%	31%	**0.001**	48%	0.34
Mean birth weight (g)		3577	3530	0.26	3273	**< 0.001**
Head circumference (cm)		34.6	34.6	0.76	34.1	**< 0.001**
Mean Apgar 5 min		8.9	8.9	0.60	8.8	0.11
Mode of birth^3^	NVB	49%	55%	0.13	75%	**< 0.001**
	Vacuum	14%	15%	0.82	11%	**< 0.001**
	Forceps	36%	30%	0.14	14%	**< 0.001**
	Breech	1%	0.3%	0.12	1%	0.41
Episiotomy rate^4^		47%	38%	**0.04**	24%	**< 0.001**
Shoulder dystocia^4^		12%	13%	0.71	5%	**< 0.001**
Instrumental level	Outlet Low cavity Mid-cavity High	(*n* = 105) 9% 29% 63% 0%	(*n* = 176) 10% 31% 60% 0%	0.08 0.71 0.60	(*n* = 4221) 10% 36% 53% 0.2%	0.44 0.12 **0.048** 1.0
Grade of OASI^5^	3a	51%	56%	0.34	55%	0.33
	3b	33%	30%	0.52	31%	0.52
	3c	7%	8%	0.66	7%	0.96
	4	7%	5%	0.24	7%	0.86
	Not coded	1%	1%	1.0	< 1%	1.0

^1^2011 data not available: table shows aggregate of 2009, 2010, 2012–2014 (*n* = 20,151 births)

^2^Parity prior to OASI birth

^3^Total RHW births: Percentage in this table is percentage of vaginal births. For all births 2009–2014 percentages were NVB 52.3%, vacuum 7.6%, forceps 9.5%, vaginal breech 0.7% and cesarean 29.9%

^4^In vaginal births

^5^As proportion of total OASI. For overall hospital population, OASI rate 2009–2014 was: 3a tear 1.5% (2.1% of vaginal births), 3b 0.8% (1.2%), 3c 0.2% (0.3%) and 4th degree 0.2% (0.3%) for a total OASI rate of 2.7% (3.8% of vaginal births)

When comparing participating women with the overall hospital population over the study time period, participants were slightly younger, much more likely to be having their first baby and gave birth at slightly longer gestation with correspondingly higher birthweight and head circumference. Compared with the overall hospital population giving birth vaginally, participants in the study were also significantly more likely to have an instrumental birth rather than an unassisted vaginal birth. They also had higher rates of episiotomy and shoulder dystocia during their birth (Table 1). This comparative data collection is taken from the full calendar years of 2009–2014 at the same hospital, providing a similar albeit slightly broader comparative sample.

Over half the participating women (108/200: 54%) were found to be experiencing an OASIACO. Of these women, 52% met the criteria for one adverse outcome (OASIACO1), 39% met the criteria for two (OASIACO2) and 9% met the criteria for all three components of the (OASIACO3).

Analysis of the OASIACO components (Table 2) revealed that 38% of women were experiencing significant ongoing bowel symptoms. The problematic bowel symptoms included either flatal incontinence or fecal urgency (occurring “sometimes” or more often) and/or fecal incontinence (occurring “occasionally” or more often). Forty-seven percent of women reported their OASI as having an ongoing impact on their daily life at the time of completing the questionnaire. Thirty-seven percent of women reported an ongoing daily life impact in three or more of the six possible categories: physical, emotional, sexual, ability to work, ability to exercise and ability to do normal daily activities. Sexual dysfunction was reported by 64% of women, with marked sexual dysfunction meeting the threshold score for the OASIACO, being identified by 10% of women.

**Table 2:** Women reporting individual adverse outcomes expressed as number (and percentage of 200 women in this overall group)

Adverse outcome	Number of women (%) reporting symptoms Total *n* = 200
Significant overall bowel symptoms	75 (38%)
Flatal incontinence or fecal urgency (“sometimes” or more)	67 (34%)
Fecal incontinence (“occasionally” or more)	38 (19%)
Impact on daily life indicated in three or more reported areas	73 (37%)
Higher than average level of sexual dysfunction score (> 5.04)	20 (10%)

These Adverse Outcomes formed the components of the OASIACO

**Fig. 2:**
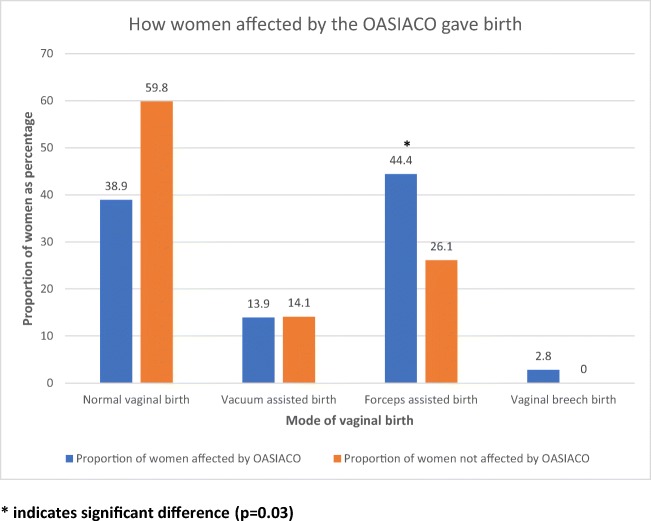
Comparing women who have been affected by the OASIACO to those not affected by the OASIACO when observing mode of birth

**Fig. 3:**
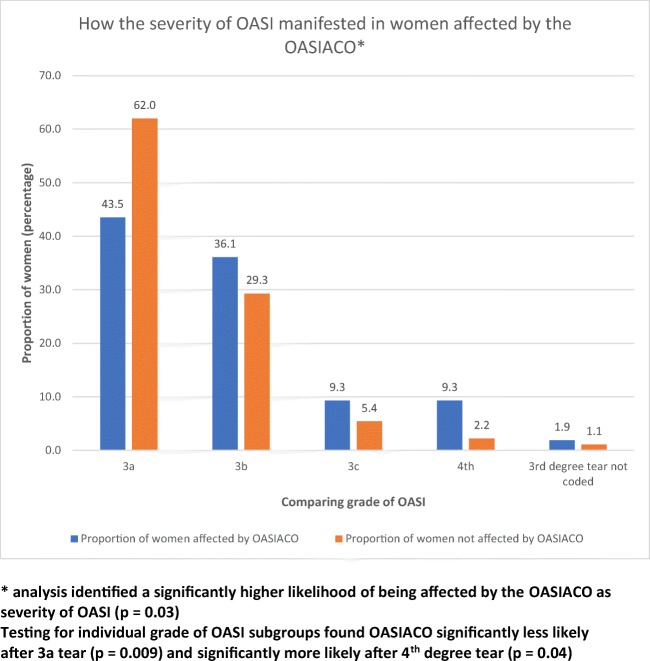
Evaluating the relationship between the severity of tear women incurred and whether they were affected by the OASIACO

Women who experienced a forceps-assisted birth were significantly more likely to have an OASIACO (*p =* 0.03; odds ratio for OASIACO if forceps-assisted versus other vaginal birth 2.3; 95% CI 1.2–4.1), with no association found between OASIACO and ventouse-assisted birth (Fig. 2). Women were also significantly more likely (*p =* 0.03) to be affected by an OASIACO if they had experienced a grade 3c or 4th degree tear compared with 3a or 3b (Fig. 3: OR for OASIACO if 3c or 4th degree tear versus 3a or 3b tear 2.8; 95% CI 1.1–6.9). Women affected by an OASIACO were more likely to report the OASI affected their future birth choices (*p* = 0.004; OR 3.3, 95% CI 1.5–7.5); if their birth choices were affected, they were more likely to meet the criteria for more than one of the component adverse outcomes. Women who reported feelings of lower levels of support (on a 5-point Likert scale) during the birth were also more likely to have an OASIACO (*p* = 0.001; OR 15.7 for ACO if reporting mostly or completely unsupported during birth versus higher levels of support, 95% CI 2.0–120.5): 16 women fell into this category. The risk of experiencing an OASIACO was not associated with maternal age, length of gestation, parity or baby’s birthweight or head circumference (all *p* > 0.15). On time period analysis, the presence of an OASIACO did not decrease with time since birth: women with OASIACO were a mean of 51 ± 15 months post-OASI versus 50 ± 15 months if no OASIACO was reported.

Regarding specific symptoms, 93 women reported their OASI had an impact on their day-to-day lives, with 19 women reporting they were “unsure.” A breakdown of ongoing reported daily life impact reveals: 92 women experience physical impact, 81 women experience impact with sexual intercourse, 68 women report an emotional impact, 60 report their ability to exercise is impacted, 46 women reported normal daily activities were affected, and 21 reported difficulty with return to work.

Further details of the specific responses from women completing the sexual function section of the questionnaire revealed that of the 169 women identifying as sexually active, 64% reported some degree of pain with intercourse and 24% reported a moderate or great degree of bother with their sexual symptoms. Thirty-one women were no longer sexually active, with 13/200 (7%) reporting their abstinence as due to pain, dryness or embarrassment. Self-reported postpartum depression or anxiety was indicated by 26% of women, with a further 16% reporting they were “unsure.” Twenty-two percent of women experienced daily or weekly urinary incontinence.

At the time of the questionnaire, 101 (51%) of the participating women had at least one more child, 19 (10%) were pregnant, and 80 (40%) had not given birth since their OASI. Of the 80 women who had no further children, 29 women indicated that their OASI impacted this decision, with 19 deciding against further children as a direct consequence of the tear.

Of the 120 women having had further children or who were pregnant, 54 (45%) indicated that their OASI had an impact on planning their following birth, with 37 (31%) having a planned cesarean delivery and 32 (27%) selecting care with a private obstetrician for their subsequent birth. Women who reported their OASI had an impact on their future birth choices were significantly more likely to choose a cesarean birth (*p* < 0.001) and to opt for private obstetrician-led care (*p* = 0.01). With respect to mode of subsequent birth, 68/101 women went on to have a vaginal birth, with 6 (9%) sustaining a second OASI.

## Discussion

This retrospective cohort has illuminated that at an average of 4 years post-OASI there is considerable ongoing morbidity. Over half the women participating (54%) reported symptoms that we propose the average individual would deem unacceptable. The reported impact to specific bowel symptoms and daily life an average of 4 years post-OASI is high and significant. Kumar et al. [13] found similar proportions of women experiencing ongoing impact on their daily life due to flatal and fecal incontinence, with 53% of their symptomatic women needing to “alter their lifestyle” because of their symptoms and 37% of women experiencing anal incontinence at a minimum of 4 years post-OASI.

A forceps-assisted birth is more likely to result in an OASIACO, but this is not so with vacuum-assisted births. This may be because women having forceps-assisted births were more likely to experience a more severe OASI or have undergone a more difficult birth, consistent with its prior identification as an OASI risk factor [14, 15]. The similar time since birth in women with and without OASIACO is notable, suggesting there is no spontaneous resolution with time of the women’s ongoing symptoms. Women were more likely to be affected by an OASIACO if they experienced a more severe grading of tear (3c or 4th), a similar finding to that of Roos et al. [1], which imparted reassurance for the use of the OASIACO as an indicator of morbidity or impairment.

The impact on subsequent birth choices is a serious and significant outcome of OASI that may be clinically noted but is lacking in the literature. The presence of an OASIACO was statistically correlated with choosing a cesarean delivery, not a more severe grade of tear alone. Analysis identified that women affected by an OASIACO were also more likely to report the OASI affected their future birth choices; if their birth choices were affected, they were more likely to have met the criteria for more than one component of the OASIACO. The finding that women with an OASIACO were significantly more likely to choose privately funded, obstetric-led care for future births may reflect the birthing woman’s desire for more control over the subsequent birth choices as well as seeking a model of continuous care with a known health care provider.

The following vaginal delivery rate of 67% (versus approximately 95% for multiparous women in general) [16] is not unexpected given the ongoing morbidity experienced. An 8.8% rate of repeat OASI is in keeping with the literature, with Antonakou et al. [17] reporting rates of 8.4%. These rates should be disclosed when counseling women on subsequent birth choices, the reality being that for one in ten of the women surveyed, they decided to have no further children because of their OASI.

Our questionnaire revealed reported postpartum depression and/or anxiety (they were not delineated) rates were at 25.5% of all women completing the questionnaire. Perinatal Anxiety and Depression Australia (PANDA) reports one in seven women experience perinatal anxiety and/or depression [18]. There is some mention of the effects of OASI on mental health in previous research [3, 19]; however, there was no information available on the rates of perinatal anxiety or depression in women affected by OASI at the time of this report. While our findings do not imply a causative link, they do present an avenue warranting further screening and follow-up of women following OASI. Post-traumatic stress disorder scoring was not part of this questionnaire, but was identified by one of the women responding, so could be an avenue for further insight.

**Fig. 4:**
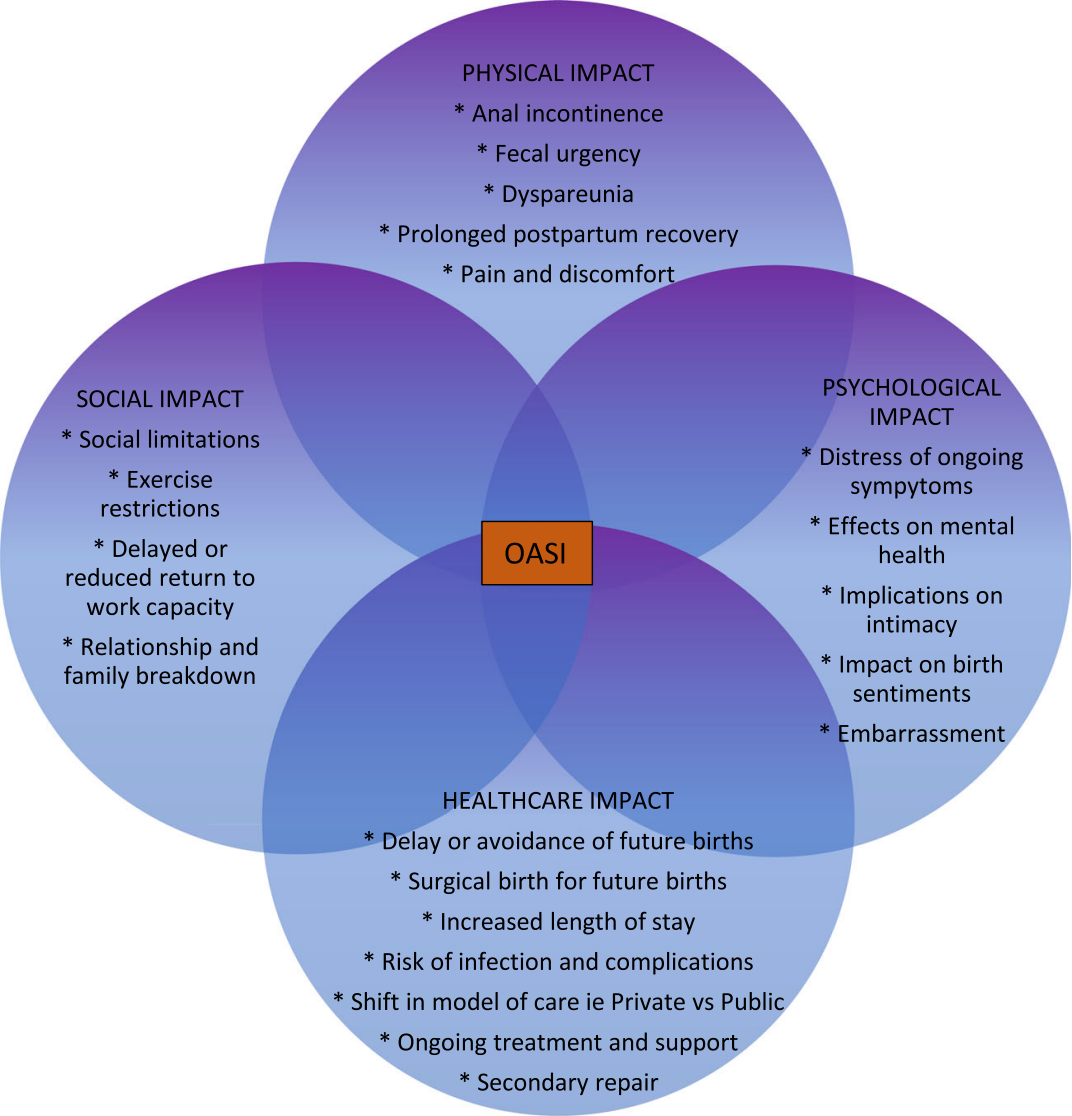
Complex web of morbidity following OASI

The impact of OASI on women is complex, so the creation of an OASIACO was an attempt to encapsulate the multifaceted nature of ongoing symptomology, as identified in the ‘web of morbidity’ (Fig. 4). The web of morbidity was mapped in an attempt to illuminate the widespread and interrelated effects an OASI can have; this helped to inform our choice of outcome measures. The components of the OASIACO included a sexual function assessment, based on the values presented in the Australian Pelvic Floor Questionnaire’s baseline evaluations with scoring above their identified average score of 5.04 [12] for sexual dysfunction as unacceptable. Only one in ten women scored above this threshold, much lower than expected, given responses to individual questions suggested a higher frequency of sexual dysfunction—with 64% of women identifying as sexually active reporting pain during intercourse. It seems that use of this threshold may have been a less adequate measure than anticipated and therefore resulted in a lower representation of women in this OASIACO category than may be considered truly representative. Therefore, the OASI impact may be even greater than suggested by the OASIACO. However, as we are not aware of any single validated questionnaire that encompasses the various aspects of OASI morbidity, we believe our questionnaire and customized OASIACO framework give at least an overview and appropriate starting point for discussions between women and their clinicians.

The correlation between having an OASIACO and feelings of being unsupported during the birth need to be interpreted with caution. Although there is evidence of better birth outcomes with high-level support during labor, including one-on-one care [20], the correlation to OASIACO in this study could also reflect an altered perception of labor and birth events following adverse outcomes rather than a causative link. The nature of this retrospective analysis will make some of this information a little less potent, but could be further explored in future prospective studies such as the Women’s Health Australasia National Collaborative on third and fourth degree tears [21].

Study limitations identified included: the proportion of women who could not be contacted because of postal contacts being no longer current was high, and this had a negative impact on the overall sample size. However, the response rate among eligible, contactable women was pleasing at 79%. It must be acknowledged that the women who did participate may have been experiencing more severe symptomology and therefore were more motivated to participate. However, there were no obvious obstetric differences between the women responding and those that did not; two attempts at contact were made to all eligible women via an alphabetical list. Not all relevant demographic data were available from the study hospital’s database, in particular ethnicity: country of birth was available; however, as this is known to be an inexact proxy for ethnicity [22], we have only reported Australian-born versus overseas-born.

Another study limitation is lack of a control group. This may have brought greater insight into the patterns of morbidity observed. As with all questionnaires involving past events, there is the limitation of recall bias (for example, regarding support during birth and regarding initial postpartum follow-up), although the majority of the questionnaire deals with current symptomatology. Self-report versus “objective” clinical measurement such as digital examination and anal manometry could also be considered a limitation. However, given that in many instances these measures have poor correlation for detecting sphincter defects, we believe that questionnaire self-report is a more appropriate way to assess medium-term OASI symptoms for the woman as an individual, as such was the study aim [23].

Our findings of 54% of women continuing to experience adverse outcomes at on average > 4 years post-OASI is incongruent with the most recent Green-Top Guidelines [24], recommending that women be told “60–80% of women are asymptomatic [in] 12 months.” It seems that either this statistic is not necessarily reflective of our particular population or perhaps our holistic longer-term follow-up approach has unmasked women previously assumed to be asymptomatic. This study’s findings suggest that physical, psychosocial and sexual symptoms of OASI last well beyond the standard follow-up period in more than half the women affected, and certainly beyond 12 months. Therefore, greater multidisciplinary and longer term care for women affected by OASI should be considered. Improved access to medical, colorectal, mental health, pelvic floor physiotherapy and sexual health care are indicated, especially prior to future birth choices.

It is recommended that increased energy and resources be allocated to implementing prevention programs in Australia, such as the perineal protection programs [25, 26], which have shown a 50% reduction in OASI rates, and a 75% reduction to 3c and fourth degree tears specifically. In recent years following the commencement of this research, a positive shift toward addressing this needs gap in recent years has occurred. In the UK, an “OASI care bundle package” has been implemented [27], led by RCOG and Royal College of Midwives, and in a selection of Australian Hospitals, the Women’s Healthcare Australasia project has also since been implemented [21]. We are confident that our data highlight the need for such programs and hope that these interventions will have a positive impact. The impact of the program on rates of OASIACO would be an avenue of future research interest.

We believe the OASIACO would be well placed as a clinical prediction tool for significant ongoing symptomology in women following an OASI incident. This in itself could lead to earlier and, possibly more meaningful, intervention and effective and efficient resource allocation in post-partum maternity services. We also believe the OASIACO could be used as a screening tool for all women in the postpartum period to assist in identifying any previously unrecognized OASI. Knowing that reported rates of OASI are actually much lower than rates determined from postpartum endoanal ultrasound [28] means that perhaps these two tools could have a powerful role to play in early and effective health care support to postpartum women.

Regarding future avenues for research, further analysis into the economic cost of having an OASI to the health care system would be worthwhile. Given the increased length of stay, surgical repair, higher rates of future cesarean delivery, ongoing multifaceted morbidity and increasing rates of OASI globally [29, 30], it follows that there would be high economic health care burden. A prospective cohort study may be another future research direction, as this was beyond the scope of this study.

## Conclusion

The overall cost of an OASI to a woman is considerable; more than half the women who sustain an OASI will have ongoing symptoms an average of 4 years post birth. The impact on daily life is substantial, as are the ramifications for the health care system.

### Electronic supplementary material

ESM 1 (DOCX 13.5 kb)

ESM 2 (DOCX 164 kb)

## Appendix

### Classification of obstetric anal sphincter injury

A table describing each classification of perineal tear that may occur during childbirth.

### The study questionnaire

The questionnaire used in this study as it would have appeared in hardcopy.

## Abbreviations

OASI: Obstetric Anal Sphincter Injury
OASIACO: Adverse Composite Outcome Score for OASI
