# Supplementary material for: What is the total impact of an obstetric anal sphincter injury? An Australian retrospective study
Source: Int Urogynecol J. 2019 Sep 16;31(3):557–66. doi: 10.1007/s00192-019-04108-3 (PMC7093361; doi:10.1007/s00192-019-04108-3)
Supplement: Supplementary file 1 — (DOCX 13.5 kb) [file 192_2019_4108_MOESM1_ESM.docx]

**S1 - Classification of Obstetric Anal Sphincter Injuries**

| Classification of Tear | Description |
| --- | --- |
| Grade 3a tear | Injury to the perineum involving the anal sphincter complex with less than 50% of external anal sphincter thickness torn |
| Grade 3b tear | Injury to the perineum involving the anal sphincter complex  with more than 50% of the external anal sphincter thickness torn |
| Grade 3c tear | Injury to the perineum involving the anal sphincter complex including both the external anal sphincter and internal anal sphincter torn |
| Grade 4 tear | Injury to the perineum involving the anal sphincter complex  (EAS and IAS) and anorectal mucosa |

Royal College of Obstetricians and Gynaecologists. The management of third- and fourth-degree perineal tears [Internet]. Green-top Guideline No. 29. London, UK; June 2015
