# Supplementary material for: What is the total impact of an obstetric anal sphincter injury? An Australian retrospective study
Source: Int Urogynecol J. 2019 Sep 16;31(3):557–66. doi: 10.1007/s00192-019-04108-3 (PMC7093361; doi:10.1007/s00192-019-04108-3)
Supplement: Supplementary file 2 — (DOCX 164 kb) [file 192_2019_4108_MOESM2_ESM.docx]

**Royal Hospital for Women OASIS follow-up questionnaire**

Thank you for agreeing to participate in this important research. Your feedback is important to us.

The following questions will be referring to your experiences surrounding your birth at The Royal Hospital for Women (RHW) between October 2009 and May 2014, during which you experienced a third or fourth degree tear.

We expect the questionnaire you have consented to undertake will take you approximately 15 minutes to complete. The questionnaire is divided into 3 parts:

**Section 1**

This section seeks to reflect on your birth experience and the amount of support you feel you received from health professionals during, and following your birth.

**Section 2**

This section explores if there have been any particular challenges in your intimate life, in particular if there are any symptoms you are experiencing during sexual intercourse.

**Section 3**

This section will focus on specific questions relating to your bowels; whether there have been any changes to your bowel function, and if so, how you are coping with this. If you have not experienced any problems with your bowel function, you will be able to indicate this also.

We understand that given the sensitive nature of some of the questions, you may be reluctant to answer all the questions. We would like to reassure you again that we have coded the questionnaires to a number to de-identify your responses. Should data be published from this research, there will be no way of identifying you, or which responses were yours. We ask that you attempt to complete all questions to the best of your knowledge as this helps us to understand your needs as accurately as possible.

After you have finished the questionnaire, if you feel that you need or would like some advice from a health professional such as Doctor, Physiotherapist, Midwife or Counsellor we would be happy to arrange a meeting to discuss your needs either in person, or over the phone. We can then take the most appropriate course of action from there.

If you have access to the internet, and would be happy to complete the questionnaire online, we invite you to do so, as it assists us with the data collection process. The online version of the questionnaire is available at the following address:

[www.surveymonkey.com/s/OASISRHW](http://www.surveymonkey.com/s/OASISRHW)

Thank you again for your participation, and please remember that we are more than happy for you to contact us if you need any assistance with completing the questionnaire.

Many thanks,

Elizabeth Evans & Clorinda Falivene

**Senior Physiotherapists - Royal Hospital for Women**

Date questionnaire completed:

**SECTION 1: Birth and the early postnatal period**

All questions in this section refer to the birth in which you experienced a 3^rd^ or 4^th^ degree tear.

**Question 1**
Following the birth, did you need to, or choose to access any health care professionals as a result of your tear?

This may include, your GP, obstetrician, midwife, physiotherapist, psychologist, counsellor etc.,

For example: you may have had an appointment to check your stitches, debrief on your birth, strengthen your pelvic floor muscles, discuss ongoing symptoms.

☐ Yes (**Go to Question 2**)

☐ No (**Go to Question 3**)

**Question 2**Could you please fill in the table below with the following:

- Which health care practitioners you visited in the period **following the birth** in which you experienced a 3^rd^ or 4^th^ degree tear – (indicate ✓ next to those applicable)
- The approximate number of visits you remember having with each health practitioner following this birth; and
- If you found those interactions helpful following this birth – (Yes/No/Somewhat)

| **Health Practitioner** | Indicate if visited? | Number of visits | Helpful?  Yes / No / Somewhat |
| --- | --- | --- | --- |
| RHW Obstetrician |  |  | Yes☐ No☐ Somewhat☐ |
| RHW birth injury clinic (OASIS clinic) |  |  | Yes☐ No☐ Somewhat☐ |
| RHW Midwife |  |  | Yes☐ No☐ Somewhat☐ |
| RHW Physiotherapist |  |  | Yes☐ No☐ Somewhat☐ |
| RHW Mental Health services |  |  | Yes☐ No☐ Somewhat☐ |
| Colorectal specialist |  |  | Yes☐ No☐ Somewhat☐ |
| St George Hospital for anal ultrasound |  |  | Yes☐ No☐ Somewhat☐ |
| Your GP |  |  | Yes☐ No☐ Somewhat☐ |
| Private Physiotherapist |  |  | Yes☐ No☐ Somewhat☐ |
| Private Psychologist |  |  | Yes☐ No☐ Somewhat☐ |
| Sexual Therapist |  |  | Yes☐ No☐ Somewhat☐ |
| Other? Please state: |  |  | Yes☐ No☐ Somewhat☐ |

NB: RHW = Royal Hospital for Women

**Please continue on to Question 4**

**Question 3**

You have indicated that you didn’t seek any further assistance from a health professional following this birth. Please indicate the single most appropriate reason why you did not seek assistance:

☐ I didn’t feel that I needed to as I recovered well after the birth

☐ I wasn’t aware of any services that could help me

☐ I was too embarrassed to discuss my symptoms and concerns

☐ I had some symptoms and knew services existed, but was very busy in the months after the birth and could never find the time to come to appointments

Other:______________________________________________________________________________________________________________

**Please continue on to Question 4**

**Question 4**

When reflecting on this birth, please indicate (✓) the response you feel is most true for you:

|  | Completely supported | Mostly supported | Somewhat supported | Mostly unsupported | Completely unsupported |
| --- | --- | --- | --- | --- | --- |
| Leading up to the birth I felt: |  |  |  |  |  |
| During the birth I felt: |  |  |  |  |  |
| During my stay in hospital I felt: |  |  |  |  |  |
| In the first 3 months post-birth I felt: |  |  |  |  |  |

**Question 5**

Reflecting on this birth again, please indicate (✓) the most appropriate response:

|  | Strongly agree | Mostly agree | Unsure | Mostly disagree | Strongly disagree |
| --- | --- | --- | --- | --- | --- |
| My memories from this childbirth experience are mostly positive: |  |  |  |  |  |
| My memories from this childbirth experience are mostly negative: |  |  |  |  |  |
| Some memories from this childbirth experience make me feel sad: |  |  |  |  |  |

**Question 6**

Did you experience postpartum depression or anxiety following this birth?

☐ Yes

☐ No

☐ Unsure

**Question 7**

Do you feel that the tear that occurred during this birth has had an impact on your day-to-day life?

☐ Yes (**Go to Q8**)

☐ No (**Go to Q9**)

☐ Unsure (**Go to Q8)**

**Question 8**

In what way(s) has it impacted?

|  | Indicate Yes or No? | Comments |
| --- | --- | --- |
| Physically |  |  |
| Emotionally |  |  |
| Ability to do normal activities |  |  |
| Ability to return to work |  |  |
| Ability to exercise |  |  |
| Sexual intercourse |  |  |
| Other (Please specify) |  |  |

**Please continue on to Q9**

**9.** Have you given birth since your third or fourth degree tear?

☐ Yes – once or more (**Go to Q10**)

☐ Not yet but currently pregnant (**Go to Q15**)

☐ No (**Go to Q18**)

***These questions are for women who have had further children***

**10.** Did your birth experience during which you incurred a 3^rd^ or 4^th^ degree tear affect your delivery choices for the following birth? (i.e. vaginal birth or caesarean)

☐ Yes

☐ No

**11.** What model of care did you chose for the following birth?

☐ Midwife led care

☐ GP shared care

☐ Private Obstetrician

☐ Public antenatal clinic

**12.** What type of birth did you **plan** **to have** in the birth following your 3^rd^ or 4^th^ degree tear?

☐ Vaginal birth

☐ Planned/Elective caesarean

**13.** What type of birth did you **actually have** in the birth following your 3^rd^ or 4^th^ degree tear?
 ☐ Vaginal birth

☐ Planned/Elective caesarean

☐ Emergency caesarean

**14.** Did you experience a 3^rd^ or 4^th^ degree tear in the following birth?

☐ Yes

☐ No

***These questions are for women currently pregnant***

**15.** Has the birth experience during which you incurred a 3^rd^ or 4^th^ degree tear, affected your planned mode of birth? (i.e. vaginal birth or caesarean)

☐ Yes

☐ No

**16.** What type of birth do you have planned?

☐ Vaginal birth

☐ Planned/Elective caesarean

☐ Unsure at this stage

**17.** What model of care have you chosen?

☐ Midwife led care

☐ GP shared care

☐ Private Obstetrician

☐ Public antenatal clinic

***These questions are for women who have had no further pregnancies***

**18.** Has your birth experience or tear, impacted on your decision to have further children?

☐ Yes

☐ No

☐ Unsure

**19.** Do you hope to have any more children in the future?

☐ Yes

☐ No

☐ Unsure

**20.** Which model of care would you choose for a future pregnancy? (please select only one option)

☐ Not applicable (do not intend any future pregnancies)

☐ Midwife led care

☐ GP shared care

☐ Private Obstetrician

☐ Unsure

**21.** What type of birth would you plan for a future pregnancy? (please select only one option)

☐ Not applicable (do not intend any future pregnancies)

☐ Vaginal birth

☐ Planned/Elective Caesarean

☐ Unsure

***For all women****:*

**Question 22**

Please feel free to add any further comments that would give us further insight into your experience or on areas we have not covered that are important to you*:*

____________________________________________________________________________________________________________

____________________________________________________________________________________________________________

­­­­­­­­­­­­­­­­­­­______________________________________________________________________________________________________________________

­­­­­­­­­­­­­­­­­­­______________________________________________________________________________________________________________________

­­­­­­­­­­­­­­­­­­­______________________________________________________________________________________________________________________

­­­­­­­­­­­­­­­­­­­______________________________________________________________________________________________________________________

**Please proceed to SECTION 2**

**SECTION 2: Sexual function**

**The following questions are taken (with permission) from the Australian Pelvic Floor Questionnaire^*^**

Please indicate the most applicable answer. Consider your experiences during the last month.

**23.** Are you sexually active?

☐ no

☐ less than once per week

☐ once or more per week

☐ daily or most days

***If you are not sexually active, please continue to answer questions 24 and 32 only***

**24.** If you are not sexually active, please tell us why:

☐ do not have a partner

☐ I am not interested

☐ my partner is unable

☐ vaginal dryness

☐ too painful

☐ embarrassment due to prolapse or incontinence

☐ other reasons: ___________________________________________

**25.** Do you have sufficient natural vaginal lubrication during intercourse?

☐ yes

☐ no

**26.** During intercourse vaginal sensation is:

☐ normal / pleasant

☐ minimal

☐ painful

☐ none

**27.** Do you feel that your vagina is too loose or lax?

☐ never

☐ occasionally

☐ frequently

☐ always

**28.** Do you feel that your vagina is too tight?

☐ never

☐ occasionally

☐ frequently

☐ always

**29.** Do you experience pain with sexual intercourse?

☐ never

☐ occasionally

☐ frequently

☐ always

**30.** Where does the pain during intercourse occur?

☐ not applicable, I do not have pain

☐ at the entrance to the vagina

☐ deep inside, in the pelvis

☐ both at the entrance and in the pelvis

**31.** Do you ever leak urine during sexual intercourse?

☐ never

☐ occasionally

☐ frequently

☐ always

**32.** How much do these sexual issues bother you?

☐ not applicable, I do not have problems

☐ not at all

☐ slightly

☐ moderately

☐ greatly

**Please proceed to SECTION 3**

**SECTION 3: Bladder and Bowel function**

Please indicate the most appropriate response for each question:

**33.** Do you ever experience any leaking from your bladder?

(e.g. with a sneeze, whilst running or jumping, or on approach to the toilet)

☐ Never
☐ Rarely (less than once per month)

☐ Sometimes (once/twice per month)
☐ Often (once or more per week)

☐ Daily

**34.** Have you experienced any changes in your bowel function since your 3^rd^ or 4^th^ degree tear?

**Please note -** this may include any of the following symptoms:

- Opening your bowels more or less often
- Needing to rush to the toilet to open your bowels
- Difficulty controlling wind or noticing the difference between wind and bowel urges
- Needing to modify your food choices or social life due to concerns about your bowels
- Difficulty controlling loose or solid bowel movements
- Bowel or wind leaking with a cough or sneeze
- Excessive or repeated wiping after opening your bowels
- Noticing smearing or staining on your underwear from your bowels

☐ Yes, I have experienced some bowel changes since my 3^rd^/4^th^ degree tear

**(Please continue onto Q35)**

☐ No, I have not experienced any bowel symptoms since my 3^rd^/4^th^ degree tear
**(You do not need to complete the following questions in this questionnaire as they relate specifically to potential issues with your bowels – please skip to the final question Q66)**

**The following questions are taken (with permission) from the Manchester Health Questionnaire^#^**

**35.** How would you describe your health at present?

☐ Very good

☐ Good

☐ Fair

☐ Poor

☐ Very poor

**36.** How much do you think your bowel problem affects your life?

☐ Not at all

☐ A little bit

☐ Moderately

☐ Quite a bit

☐ Extremely

**We would like to know what your bowel problems are and how much they affect you?**

**37.** How often do you have a strong desire to move your bowel, which makes you rush to the toilet?

☐ Never

☐ Occasionally

☐ Sometimes

☐ Most of the time

☐ All of the time

**38.** How often do your bowels leak when coughing or sneezing?

☐ Never

☐ Occasionally

☐ Sometimes

☐ Most of the time

☐ All of the time

**39.** How often do your bowels leak when walking?

☐ Never

☐ Occasionally

☐ Sometimes

☐ Most of the time

☐ All of the time

**40.** Do your bowels leak during the rest of the day or night?

☐ Never

☐ Occasionally

☐ Sometimes

☐ Most of the time

☐ All of the time

**41.** Do you have difficulty wiping clean after you have opened your bowels?

☐ Never

☐ Occasionally

☐ Sometimes

☐ Most of the time

☐ All of the time

**42.** Do you have difficulty controlling wind?

☐ Never

☐ Occasionally

☐ Sometimes

☐ Most of the time

☐ All of the time

**43.** Is there leakage from your bowels that is loose?

☐ Never

☐ Occasionally

☐ Sometimes

☐ Most of the time

☐ All of the time

**44.** Is there leakage from your bowels that is solid?

☐ Never

☐ Occasionally

☐ Sometimes

☐ Most of the time

☐ All of the time

**45.** How often do you move your bowels during the day?

☐ Not every day

☐ 1-2

☐ 3-4

☐ 5-6

☐ 7 or more

**46.** Do your bowels leak during or after sexual intercourse?

☐ Never

☐ Occasionally

☐ Sometimes

☐ Most of the time

☐ All of the time

☐ Not sexually active

If you are not sexually active, is it because: you are not in a sexual relationship ☐

your bowel problem makes intercourse impossible ☐

**How much does the bowel problem you described affect you?**

**Role limitations**

**47.** Does your bowel problem affect you doing jobs within the home?

☐ Never

☐ Rarely

☐ Sometimes

☐ Often

☐ Always

**48.** Does your bowel problem affect your job, or your normal daily activities outside the home?

☐ Never

☐ Rarely

☐ Sometimes

☐ Often

☐ Always

**Physical/Social limitations**

**49.** Does your bowel problem affect your ability to travel?

☐ Never

☐ Rarely

☐ Sometimes

☐ Often

☐ Always

**50.** Does your bowel problem affect your physical activities (e.g. going for a walk, running, sport, gym etc.)?

☐ Never

☐ Rarely

☐ Sometimes

☐ Often

☐ Always

**51.** Does your bowel problem limit your social life?

☐ Never

☐ Rarely

☐ Sometimes

☐ Often

☐ Always

**52.** Does your bowel problem limit your ability to see and visit friends?

☐ Never

☐ Rarely

☐ Sometimes

☐ Often

☐ Always

**Personal relationships**

**For questions 53-65: if not applicable, please leave indicate** (✓) **‘Never’**

**53.** Does your bowel problem affect your relationship with your partner?

☐ Never

☐ Rarely

☐ Sometimes

☐ Often

☐ Always

**54.** Does your bowel problem affect your sex life?
☐ Never
☐ Rarely
☐ Sometimes
☐ Often
☐ Always

**55.** Does your bowel problem affect your family life?

☐ Never

☐ Rarely

☐ Sometimes

☐ Often

☐ Always

**Emotions**

**56.** Does your bowel problem make you feel depressed?

☐ Never

☐ Rarely

☐ Sometimes

☐ Often

☐ Always

**57.** Does your bowel problem make you feel anxious or nervous?

☐ Never

☐ Rarely

☐ Sometimes

☐ Often

☐ Always

**58.** Does your bowel problem make you feel bad about yourself?

☐ Never

☐ Rarely

☐ Sometimes

☐ Often

☐ Always

**Sleep/energy**

**59.** Does your bowel problem affect your sleep?

☐ Never

☐ Rarely

☐ Sometimes

☐ Often

☐ Always

**60.** Does your bowel problem make you feel worn out and tired?

☐ Never

☐ Rarely

☐ Sometimes

☐ Often

☐ Always

**Do you do any of the following? If so, how much?**

**61.** Wear pads to keep clean?

☐ Never

☐ Rarely

☐ Sometimes

☐ Often

☐ Always

**62.** Be careful of how much food you eat?

☐ Never

☐ Rarely

☐ Sometimes

☐ Often

☐ Always

**63.** Change your underclothes because they get dirty?

☐ Never

☐ Rarely

☐ Sometimes

☐ Often

☐ Always

**64.** Worry in case you smell? (because of a bowel problem)

☐ Never

☐ Rarely

☐ Sometimes

☐ Often

☐ Always

**65.** Get embarrassed because of your bowel problem?

☐ Never

☐ Rarely

☐ Sometimes

☐ Often

☐ Always

**66.** In this space please feel free to make any comments important to you, which have not been covered.

**THANK YOU FOR TAKING THE TIME TO ANSWER THESE QUESTIONS.**

**YOUR INPUT IS GREATLY APPRECIATED!**

# Bugg et al (2001), A new condition-specific health-related quality of life questionnaire for the assessment of women with anal incontinence. *British Journal of Obstetrics and Gynaecology*, 108, 1057-1067.

* Baessler et al (2010), A validated self-administered female pelvic floor questionnaire. *International Urogynecology Journal*, 21, 163-172
